# Selective Wettability Membrane for Continuous Oil−Water Separation and In Situ Visible Light‐Driven Photocatalytic Purification of Water

**DOI:** 10.1002/gch2.202000009

**Published:** 2020-04-16

**Authors:** Mohammadamin Ezazi, Bishwash Shrestha, Sun‐I. Kim, Bora Jeong, Jerad Gorney, Katie Hutchison, Duck Hyun Lee, Gibum Kwon

**Affiliations:** ^1^ Department of Mechanical Engineering University of Kansas Lawrence Kansas 66045 USA; ^2^ Green Materials and Process Group Korea Institute of Industrial Technology Ulsan 44413 Republic of Korea

**Keywords:** annealing, iron‐doped titania, oil−water separation, photocatalysis, thermal sensitization

## Abstract

Membrane‐based technologies are attractive for remediating oily wastewater because they are relatively energy‐efficient and are applicable to a wide range of industrial effluents. For complete treatment of oily wastewater, removing dissolved contaminants from the water phase is typically followed by adsorption onto an adsorbent, which complicates the process. Here, an in‐air superhydrophilic and underwater superoleophobic membrane‐based continuous separation of surfactant‐stabilized oil‐in‐water emulsions and in situ decontamination of water by visible‐light‐driven photocatalytic degradation of dissolved organic contaminants is reported. The membrane is fabricated by utilizing a thermally sensitized stainless steel mesh coated with visible light absorbing iron‐doped titania nanoparticles. Post annealing of the membrane can enhance the adhesion of nanoparticles to the membrane surface by formation of a bridge between them. An apparatus that enables continuous separation of surfactant‐stabilized oil‐in‐water emulsion and in situ photocatalytic degradation of dissolved organic matter in the water‐rich permeate upon irradiation of visible light on the membrane surface with greater than 99% photocatalytic degradation is developed. The membrane demonstrates the recovery of its intrinsic water‐rich permeate flux upon continuous irradiation of light after being contaminated with oil. Finally, continuous oil−water separation and in situ water decontamination is demonstrated by photocatalytically degrading model toxins in water‐rich permeate.

## Graphical abstract

An apparatus enables continuous separation of surfactant‐stabilized oil‐in‐water emulsion and in situ photocatalytic degradation of organic matter in the water‐rich permeate upon visible light irradiation. The membrane demonstrates recovery of its intrinsic water‐rich permeate flux upon continuous light irradiation after contamination with oil. The predictions using the first‐order kinetic model match well with the experimental data of photocatalytic degradation.

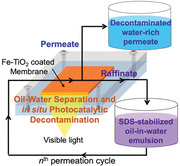

## 1 Introduction

Membrane‐based technologies are attractive in oily wastewater treatment because they typically do not require chemical additives, thermal inputs, or regeneration of spent media.^[^ ^1^, ^2^, ^3^, ^4^ ^]^ Despite recent advances, membranes are fundamentally limited by their inability to remove the dissolved contaminants from the permeate (e.g., water) phase.^[^ ^5^, ^6^ ^]^ This is because membranes separate contaminants based on their sizes.^[^ ^7^ ^]^ When the membrane pore size is larger than the contaminant, it readily passes through along with the permeate phase.^[^ ^8^ ^]^ Further, the dissolved contaminant can adsorb to the membrane surface and pore walls, resulting in fouling.^[^ ^9^ ^]^ When a membrane is fouled, it not only reduces the permeate flux and purity but also leads to shortened membrane lifespan and thereby an increase of the operating cost.^[^ ^10^, ^11^, ^12^ ^]^ Therefore, membrane‐based technologies typically involve a prefiltration step to remove suspended and dissolved contaminants.^[^ ^13^, ^14^ ^]^ In addition, membranes are subjected to periodic backwashing (or forward flushing) and chemical treatment to clean the membrane surface and pores.^[^ ^15^, ^16^ ^]^ However, implementing these methods incurs process downtime, and can cause membrane damage and degradation over time, which decrements the membrane's performance.^[^ ^17^ ^]^

Modulating the membrane's wettability enhances its resistance to fouling by oil.^[^ ^18^, ^19^ ^]^ We^[^ ^20^, ^21^, ^22^ ^]^ and others^[^ ^23^, ^24^, ^25^ ^]^ have demonstrated that hydrophilic (i.e., water contact angle, θ*_water_ < 90°) or superhydrophilic (i.e., θ*_water_ ≈ 0°) membranes allow water to permeate through. These membranes can repel the oil phase in air or underwater. Further, they prevent adsorption of organic contaminants by forming a thin water film on the surface, which enables oil−water separation without a decline of flux.

Recently, such membranes with selective wettability (i.e., hydrophilic and in air or underwater oleophobic) have been incorporated with a photocatalytic semiconductor (e.g., TiO_2_,^[^ ^26^ ^]^ α‐Fe_2_O_3_,^[^ ^27^ ^]^ WO_3_,^[^ ^28^ ^]^ BiVO_4_,^[^ ^29^ ^]^ β‐FeOOH,^[^ ^30^ ^]^ g‐C_3_N_4_,^[^ ^31^ ^]^ and CuWO_4_@Cu_2_O^[^ ^32^ ^]^), which allows for catalytic degradation of the organic contaminants dissolved in the water‐rich permeate upon irradiation of light. Photocatalytic materials generate electron hole (e^−^‐h^+^) pairs upon irradiation of light with an energy greater than their bandgap energy (*E*_g_).^[^ ^33^ ^]^ The electrons and holes react with ambient oxygen or water molecules and produce highly reactive radicals such as hydroxyl,^[^ ^34^, ^35^, ^36^ ^]^ superoxide anion,^[^ ^37^ ^]^ and peroxide.^[^ ^38^ ^]^ These radicals oxidize (or reduce) the organic contaminants either dissolved in the water‐rich permeate or on the membrane, which results in decontamination of the permeate and membrane cleaning.^[^ ^39^ ^]^

Herein, we developed an in‐air superhydrophilic and underwater superoleophobic membrane capable of separating surfactant‐stabilized oil‐in‐water emulsions and in situ decontamination of the water‐rich permeate by photocatalytic degradation of dissolved organic contaminants upon visible light irradiation. The membrane was fabricated by utilizing thermally sensitized stainless steel mesh coated with iron (Fe) doped titania (TiO_2_) nanoparticles (Fe−TiO_2_). Fe−TiO_2_ is chosen because it can photocatalytically degrade a variety of organic compounds including phenols,^[^ ^40^ ^]^ acetaldehyde,^[^ ^41^ ^]^ oxalic acid,^[^ ^42^ ^]^ and organic dyes^[^ ^43^ ^]^ upon visible light irradiation. We showed that post‐annealing increased the adhesion force of Fe−TiO_2_ nanoparticles to the membrane by the formation of a fusion‐induced bridge between them. We engineered a cross‐flow apparatus that enables continuous oil−water separation and in situ photocatalytic degradation of the dissolved contaminants in the water‐rich permeate upon visible light irradiation. Finally, we demonstrated complete separation of a surfactant‐stabilized oil‐in‐water emulsion and photocatalytic degradation of toxins such as dioxin and permethrin by utilizing the apparatus. We envision that our separation methodology can offer a wide range of potential applications including petroleum refining, wastewater treatment, and oil spills clean‐up.

## 2 Results and Discussion

**Figure 1:**
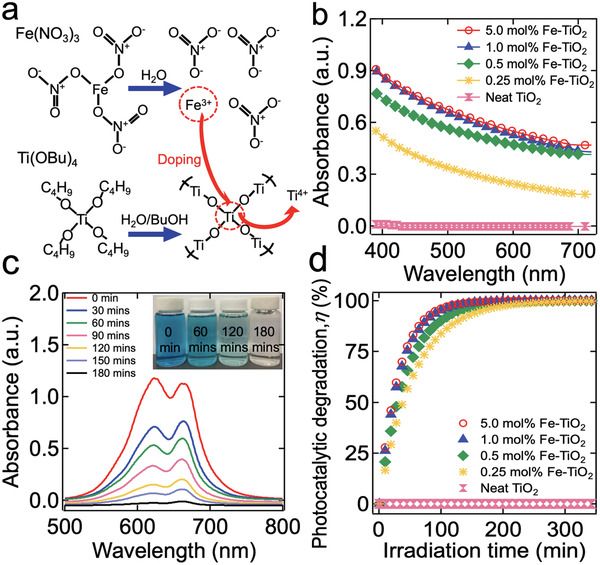
a) Schematic illustrating the synthesis of Fe‐doped TiO_2_ nanoparticles by utilizing titanium butoxide (Ti(OBu)_4_) and ferric nitrate (Fe(NO_3_)_3_) as TiO_2_ and Fe^3+^ precursors, respectively. b) UV−vis absorption spectra of Fe−TiO_2_ with varied Fe doping concentration. A neat TiO_2_ absorption spectrum is also provided for comparison. c) UV−vis absorption spectra of solvent blue dye dissolved in water (*C*_o_ = 0.02 mg mL^−1^) subjected to visible light‐driven photocatalytic degradation by Fe−TiO_2_. Inset: A photograph showing the water dissolved with solvent blue dye subjected to visible light irradiation for 60, 120, and 180 min. d) The photocatalytic degradation (η) of solvent blue dye in water by Fe−TiO_2_ with varied doping concentration as a function of the visible light irradiation time.

Iron (Fe)‐doped TiO_2_ (Fe‐TiO_2_) nanoparticles (average diameter ≈ 20 nm) were synthesized by the coprecipitation method using titanium butoxide (Ti(OBu)_4_) and ferric nitrate (Fe(NO_3_)_3_) as titania (TiO_2_) and ferric ion (Fe^3+^) precursors, respectively (**Figure** **1**a) (see Experimental Section and Section S1, Supporting Information). During coprecipitation, the alkoxide end group of Ti(OBu)_4_ is hydrolyzed in the basic solvent (1 m NaOH in water) resulting in the formation of titanium hydroxide groups. Subsequently, the hydroxide groups undergo the condensation reaction which results in the formation of titanium dioxide (TiO_2_) while Fe^3+^ can substitute titanium ion (Ti^4+^) in the TiO_2_ lattice. The ionic radius of Ti^4+^ (0.74 Å) is close to that of Fe^3+^ (0.69 Å) which makes their substitution straightforward.^[^ ^40^ ^]^ The Fe^3+^ sites serve as traps for photogenerated electron−hole pairs, which suppresses their recombination.^[^ ^44^ ^]^ Further, the Fe^3+^ dopant in TiO_2_ lattice can inhibit the undesired phase transformation fromanatase to rutile at an elevated temperature which can decrement the photocatalytic activity of TiO_2_.^[^ ^41^ ^]^ The resulting Fe−TiO_2_ exhibits a bandgap energy of ≈2.67 eV^[^ ^45^ ^]^ with lower electron−hole pairs recombination rate.^[^ ^40^ ^]^ Therefore, Fe−TiO_2_ can absorb a broad range of the visible light spectrum (i.e., 390 nm < λ < 750 nm) whereas neat TiO_2_ (i.e., undoped) absorbs only in the ultraviolet spectrum (i.e., λ < 390 nm) (Figure 1b).

Fe−TiO_2_ degrades organic compounds upon visible light irradiation (i.e., photocatalysis). Figure 1c shows the ultraviolet−visible (UV−Vis) absorption spectra of a solvent blue 38 dye dissolved aqueous solution (concentration = 0.02 mg mL^−1^) subjected to visible light‐driven photocatalytic degradation by Fe−TiO_2_ (Experimental Section). The intensity of the incident light was 150 mW cm^−2^. After 180 min of irradiation, the solution became almost colorless indicating that nearly all dye molecules were degraded. We demonstrated that Fe−TiO_2_ with a higher Fe^3+^ doping concentration leads to more rapid photocatalysis of dye molecules. Figure 1d shows the photocatalytic degradation (η) of solvent blue dye as a function of irradiation time. Here η is defined as η = (1 − *C*_n_/*C*_o_) × 100; *C*_o_ and *C*_n_ are the initial concentration of solvent blue dye and the concentration at time *n*, respectively. It illustrates that when TiO_2_ is doped with Fe^3+^ with a concentration greater than 1.0 mol% with respect to TiO_2_, the photocatalysis rates remain almost unchanged. Thus, we utilized TiO_2_ doped with Fe^3+^ with a doping concentration of 1.0 mol% with respect to TiO_2_ throughout this report. We also demonstrated that a higher intensity of the incident light can result in a more rapid photocatalytic degradation of solvent blue dye (Section S2, Supporting Information).

**Figure 2:**
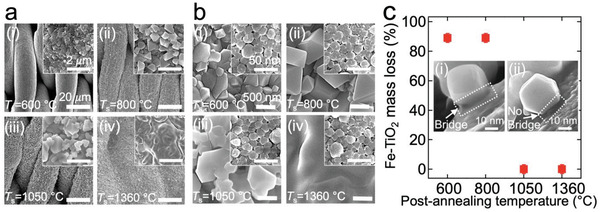
a) Scanning electron microscopy (SEM) images of SS 316 mesh sensitized at varied temperatures (*T*_s_), (i) *T*_s_ = 600 °C, (ii) *T*_s_ = 800 °C, (iii) *T*_s_ = 1050 °C, and (iv) *T*_s_ = 1360 °C, respectively. Insets show high magnification SEM images of the feature. b) SEM images of Fe−TiO_2_ coated membranes annealed at varied temperatures, (i) 600 °C, (ii) 800 °C, (iii) 1050 °C, and (iv) 1360 °C, respectively. Insets show high magnification SEM images of the membrane surface coated with Fe−TiO_2_ nanoparticles. c) The mass loss of the membranes after the standard Tape Peel off test. Inset: SEM images show (i) a bridge between the Fe−TiO_2_ nanoparticle and the underlying SS 316 steel mesh surface annealed at a temperature close to stainless steel's annealing temperature (*T*_a_ = 1040 °C) and (ii) no bridge on the mesh annealed at temperature significantly lower than the stainless steel's annealing temperature.

To improve the utility of our visible light‐responsive photocatalytic Fe−TiO_2_ nanoparticles for practical applications such as wastewater treatment, we fabricated a membrane by coating a stainless steel (SS) 316 Twill Dutch weave mesh (nominal pore size = 2 µm, Experimental Section). The mesh firstly underwent heat treatment at *T* = 1050 °C for 900 min (Experimental Section). When stainless steel is subjected to a temperature close to its annealing temperature (*T*_a_ ≈ 1040 °C for SS316^[^ ^46^, ^47^ ^]^), it undergoes chromium depletion at its grain boundaries, which results in the evolution of surface roughness (i.e., thermal sensitization).^[^ ^46^, ^48^ ^]^ We demonstrated that the sensitizing temperature (*T*_s_) affects the surface roughness. When *T*_s_ is far below *T*_a_ (e.g., *T*_s_ = 600 °C), it resulted in a finer surface texture with less roughness (**Figure** **2**ai). The surface became rougher with a coarser texture at *T*_s_ = 800 °C (Figure 2aii). When *T*_s_ is 1050 °C, a hierarchical surface texture (i.e., coarser texture with finer texture possessing bead‐like geometry) was formed (Figure 2aiii). Such a temperature‐dependent evolution of surface texture can be attributed to the selective depletion of chromium and enrichment of iron at the grain boundaries.^[^ ^48^, ^49^ ^]^ When the *T*_s_ (*T*_s_ = 1360 °C) is close to the melting temperature (i.e., *T*_m_ = 1370 °C for SS316^[^ ^46^, ^47^ ^]^), the hierarchical texture disappeared and the surface became coarser (Figure 2aiv). This can be attributed to the merging of neighboring micro‐ and nanofeatures caused by grain growth at a temperature close to the melting point. In addition to sensitizing temperature, we demonstrated that sensitizing time can affect the surface texture and roughness. We found that a sensitizing period of 900 min was optimal for the uniform coverage of surface features (Section S3, Supporting Information). Further, thermal sensitization results in a decrease of nominal pore size of themesh due to the evolution of the surface texture. We characterized the nominal pore size by utilizing filter retention analysis^[^ ^50^ ^]^ (Experimental Section). The values of root mean square (*R*_rms_) roughness, average feature size (φ), and the nominal pore size of meshes subjected to thermal sensitization at varied temperatures are listed in **Table** **1**.

**Table 1:** List of the values of *R*_rms_, feature size, and nominal pore size of the membrane subjected to thermal sensitization at varied temperature

*T*_s_ [°C]	*R*_rms_ [µm]	φ [µm]	Nominal pore size [µm]
600	0.63 ± 0.03	0.54 ± 0.08	0.75
800	1.23 ± 0.15	1.03 ± 0.09	0.50
1050	3.75 ± 0.21	0.51 ± 0.07 (finer texture) 1.29 ± 0.10 (coarser texture)	0.40
1360	0.34 ± 0.02	2.09 ± 0.11	0.20

The sensitized SS316 meshes were spray‐coated with Fe−TiO_2_ nanoparticles followed by annealing for 60 min (Experimental Section). Please note that the annealing temperature (*T*_a_) was the same as that at which the mesh was sensitized. Scanning electron microscopy (SEM) images indicate that the SS316 mesh surfaces were uniformly coated with Fe−TiO_2_ without any aggregation (Figure 2b).

Practical application of membranes often requires good mechanical integrity. This becomes nontrivial when membranes are subjected to a high shear force by the feed stream, which often causes the delamination of the coating from the membrane surface. We evaluated the adhesion force of Fe−TiO_2_ nanoparticles to the membrane surface by utilizing the standard ASTM D3359 Tape Peel off test^[^ ^51^ ^]^ (Experimental Section). The mass of the membrane after the test was measured by utilizing a high precision scale and compared with that of the as‐prepared membrane. The results showed that the mass of as‐prepared membrane and that of the membrane after the test were almost unchanged (i.e., 2296.17 and 2296.16 mg, respectively) (Figure 2c). This can be attributed to a fusion‐induced bridge that is formed during the annealing process (Figure 2c, inset (i)). The bridge between Fe−TiO_2_ and SS316 develops at a temperature close to the annealing temperature (*T*_a_ ≈ 1040 °C^[^ ^46^, ^47^ ^]^) of SS316 at which iron (Fe) atoms in the SS316 diffuse to the surface and form a meniscus with the contacting Fe−TiO_2_. Subsequently, it recrystallizes and forms a bridge. In contrast, a membrane subjected to a lower annealing temperature (e.g., *T*_a_ = 600 or 800 °C) showed ≈90% by mass delamination of Fe−TiO_2_ nanoparticles during the peel‐off test due to the lack of such a bridge (Figure 2c, inset (ii)).

**Figure 3:**
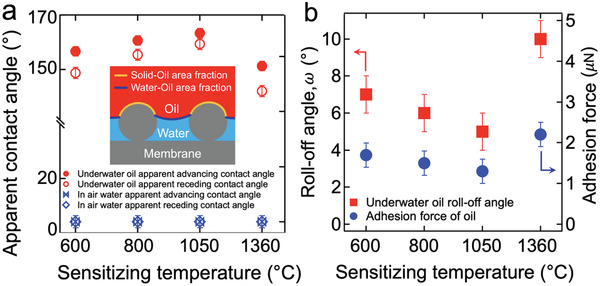
a) Measured advancing and receding contact angles for water in air and those for oil on the membrane submerged in water. Inset: Schematic illustrating composite interface of solid (membrane)−oil−water. b) Measured roll‐off angles and adhesion force of oil (*n*‐hexadecane) droplet on membranes submerged in water.

We characterized the wettability of the membranes by measuring the contact angles for water and oil (*n*‐hexadecane, γ_lv_ = 27.5 mN m^−1^). The advancing and receding contact angles (θ*_adv_ and θ*_rec_) for water are zero on all membranes (**Figure** **3**a). Such an in‐air superhydrophilic membrane demonstrates oleophobicity (i.e., oil contact angle, θ*_oil_ > 90°) or superoleophobicity (i.e., θ*_oil_ > 150°) when submerged in water. The measured θ*_adv_ and θ*_rec_ for oil are 160°±1 and 157°±2 on the membrane sensitized at *T*_s_ = 1050 °C while those on the membranes sensitized at *T*_s_ = 600 °C and 800 °C are 155°±1°, 149°±2° and 158°±1°, 154°±2°, respectively (Figure 3a). The higher underwater oil contact angles on membranes sensitized at 1050 °C compared to those on membranes sensitized at a lower temperature is attributed to a reduced area fraction of solid−oil (i.e., an increase of oil−water area fraction, Section S4, Supporting Information). Thus, we inferred that an oil droplet can be readily removed from the membrane exhibiting a reduced solid−oil area fraction. The roll‐off angles (ω, i.e., the minimum angle by which the membrane must be tilted for a droplet to roll off^[^ ^52^ ^]^) for an oil droplet on the membrane submerged in water were measured. The roll‐off angles are 7°±1°, 6°±1°, 5°±1°, and 10°±1° on the membranes sensitized at *T*_s_ = 600, 800, 1050, and 1360 °C, respectively (Figure 3b). Further, the adhesion force of an oil droplet to the membrane submerged in water was measured by utilizing a high‐precision microelectromechanical system (Experimental Section). The results showed 1.7 ± 0.3 μN, 1.5 ± 0.2 μN, 1.3 ± 0.2 μN, and 2.2 ± 0.5 μN on the membranes sensitized at 600, 800, 1050, and 1360 °C, respectively (Figure 3b). Therefore, a membrane sensitized at 1050 °C is expected to exhibit high resistance to oil fouling. The measured underwater roll‐off angles and adhesion force for oils with varied surface tension values on our membranes are provided in Section S5, Supporting Information.

**Figure 4:**
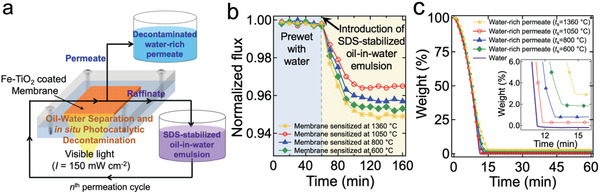
a) Schematic illustrating the continuous separation apparatus that enables continuous separation of oil−water mixture and in situ decontamination of water‐rich permeate by photocatalysis. b) The normalized flux of the time‐dependent water‐rich permeate through the membrane mounted to the apparatus. The membrane was prewetted by DI water for 60 min followed by sodium dodecyl sulfate (SDS) stabilized oil‐in‐water emulsion (5:95 v:v *n*‐hexadecane:water). c) Thermogravimetric analyses (TGA) data of the water‐rich permeate through the membranes sensitized at varied sensitizing temperatures. Inset: Zoomed‐in TGA data demonstrating varied oil concentrations.

Our membrane's selective wettability (i.e., in‐air superhydrophilicity and underwater superoleophobicity), mechanical integrity, and visible light‐driven photocatalytic degradation capability makes it suitable for separating oil−water mixtures and decontaminating the water‐rich permeate upon irradiation by light. To demonstrate this, we first engineered an apparatus that enables continuous separation of oil−water mixture and in situ photocatalytic decontamination of water permeate (**Figure** **4**a and Experimental Section). The feed oil−water mixture dissolved with organic pollutants is pumped out from the feed storage tank and introduced to the cell at which the membrane is mounted. While the feed oil−water mixture flows through the membrane, the water‐rich permeate is collected while the raffinate is reintroduced to the feed storage tank for further separation. During operation, the membrane surface is continuously irradiated with a constant light intensity.

The membrane was first soaked with deionized (DI) water for at least 60 min before being subjected to oil−water mixture. This prewet process results in a formation of a thin water film on the membrane surface, which is used to maintain underwater oleophobicity. The flux of DI water during the prewet process remained almost constant (Figure 4b). Subsequently, the feed sodium dodecyl sulfate (SDS) stabilized *n*‐hexadecane‐in‐water emulsion (5:95 v:v, Experimental Section) dissolved with solvent blue dye (concentration = 0.02 mg mL^−1^) was introduced. The transmembrane pressure (TMP, i.e., the pressure across the membrane) was maintained at 2.0 psi. The water‐rich permeate was collected in a separate container while the raffinate was reintroduced to the feed storage tank. We collected a small quantity (≈0.01 mL) of the permeate every 10 min for the thermogravimetric analysis (TGA). The flux of the water‐rich permeate gradually decreases over time (Figure 4b) due to oil fouling, which clogs the membrane pores. We found that the membranes sensitized at different temperature demonstrated varied flux decline. For example, the membrane sensitized at *T*_s_ = 1050 °C showed about 3% decrease of the flux compared to its inherent value while that sensitized at *T*_s_ = 1360 °C showed ≈5% decrease. We attribute this to their different adhesion forces and roll‐off angles for the oil droplet (see Figure 3b).

Despite the decrease of the flux for water‐rich permeate over time, the separation efficiency (ξ_separation_) remained very high (>97%) for all membranes (Figure 4c). The separation efficiency was calculated by ξ_separation_ = (1 − *C*_f_/*C*_i_) × 100, where *C*_i_ and *C*_f_ are the concentrations of oil in the feed oil‐in‐water emulsion and that in the water‐rich permeate, respectively.^[^ ^53^, ^54^, ^55^ ^]^ The oil concentration was determined by TGA (Experimental Section). We demonstrated that separation efficiency is directly related to a breakthrough pressure (*P*_b_; i.e., the minimum pressure that forces a liquid to permeate through the porous membrane) for oil to the membrane (Section S6, Supporting Information). It is worth noting that the separation efficiency was unaffected by the oil droplet size in the feed emulsion (Section S7, Supporting Information) because the membrane pore size (i.e., 0.20−0.75 µm, see Table 1) is significantly smaller than that of the oil droplet in the feed emulsion (i.e., diameter (*d*) = 1–6 µm, Section S7, Supporting Information).

**Figure 5:**
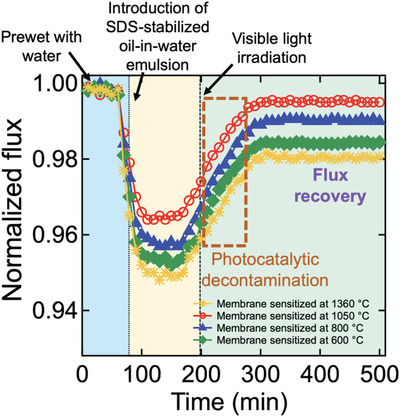
The normalized flux of the water‐rich permeate through the membranes irradiated by visible light (Intensity = 150 mW cm^−2^) during the separation of sodium dodecyl sulfate (SDS) stabilized oil‐in‐water emulsion (5:95 v:v *n*‐hexadecane:water).

We demonstrated that our membrane recovers the flux of the water‐rich permeate nearly 99% upon irradiation of visible light while it was continuously separating surfactant‐stabilized oil‐in‐water emulsion (**Figure** **5**). For example, the membrane sensitized at *T*_s_ = 1050 °C exhibited a water‐rich permeate flux of ≈304 L m^−2^ h^−1^ right after the feed SDS‐stabilized oil‐in‐water emulsion was introduced. The flux gradually decreased and reached ≈289 L m^−2^ h^−1^ after 120 min. Upon the irradiation of visible light on the membrane surface (intensity = 150 mW cm^−2^), the water‐rich permeate flux started to increase and reached ≈303 L m^−2^ h^−1^ after 150 min of irradiation while it continuously separated the oil‐in‐water emulsion. For comparison, the membranes sensitized at *T*_s_ = 600, 800, and 1360 °C demonstrated 98.2, 98.8, and 98.0% recovery of the water‐rich permeate flux compared to those obtained by utilizing as‐prepared (i.e., nonfouled) ones.

**Figure 6:**
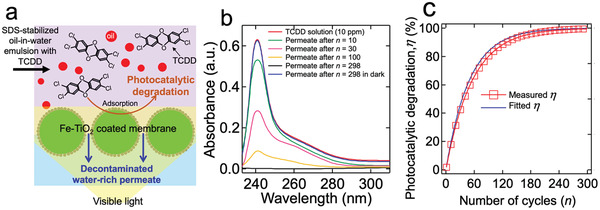
a) Schematic illustrating separation of oil‐in‐water emulsion and in situ photocatalytic degradation of TCDD dissolved in the water‐rich permeate upon irradiation of light. b) The UV−vis absorption spectra of TCDD dissolved water‐rich permeate as a function of repeated permeation cycles. c) Plots of the measured and the fitted photocatalytic degradation of TCDD as a function of permeation cycles.

Finally, we demonstrated continuous separation of surfactant‐stabilized oil‐in‐water emulsion and in situ decontamination of the water‐rich permeate by photocatalytic degradation of the dissolved organic pollutant (**Figure** **6**a). We utilized 2,3,7,8‐tetrachlorodibenzodioxin (TCDD) as a model pollutant. TCDD is one of the most prevailing dioxin compounds found in the groundwater and surface water.^[^ ^56^, ^57^ ^]^ A total of 0.5 L of an SDS‐stabilized *n*‐hexadecane‐in‐water emulsion (5:95 v:v) dissolved with TCDD (10 parts per million (ppm) with respect to water) was continuously fed to the apparatus with a flowrate of 1.0 L min^−1^. The membrane surface was continuously irradiated by visible light with an intensity of 150 mW cm^−2^. It is considered one cycle when all the feed emulsion passes through the membrane. At each cycle, the water‐rich permeate is reintroduced to the feed storage tank for further decontamination. A small quantity (≈0.01 mL) of the permeate was collected to analyze the concentration of TCDD in every 30 cycles by utilizing UV−vis spectroscopy. We repeated the cycles until all TCDD was completely degraded from the feed emulsion. Figure 6b shows the UV−vis absorption spectra of the water‐rich permeate as a function of cycles. We determined the TCDD concentration by calculating the area underneath the absorption spectra with a characteristic peak (λ ≈ 240 nm)^[^ ^58^ ^]^ and compared it with the calibration curves (Section S8, Supporting Information). The results showed that the TCDD concentration becomes ≈1.2 ppm after 100 cycles and it eventually becomes <0.1 ppm after *n* = 298 cycles. This is due to the photocatalytic degradation of TCDD dissolved in the water‐rich permeate while it passes through the membrane. Please note that the TCDD concentration in the water‐rich permeate remains almost unchanged (≈10 ppm) after 298 cycles when the membrane was in dark (Figure 6b). Figure 6c shows the photocatalytic degradation (η) as a function of permeation cycles, which is defined as η = (1 − *C*_n_/*C*_o_) × 100, where *C*_o_ and *C*_n_ are the concentrations of TCDD in the feed and that in the water‐rich permeate after *n* ^th^ cycle, respectively.

Photocatalytic degradation of TCDD is described by the first order kinetics, given by:^[^ ^59^ ^]^

(1) $$C_{\text{n}}=C_{\text{o}}{\text{e}}^{-k\tau }$$

where κ is a rate constant and τ the residence time of the water‐rich permeate in the membrane. The residence time (τ) is calculated by:^[^ ^60^ ^]^

(2) $$\tau =nV_{\text{r}}\text{/}J_{\text{w}}A_{\text{s}}$$

where *V*_r_ is the volume of Fe−TiO_2_ nanoparticles coated on the membrane, *J*_w_ the water‐rich permeate flux, *A*_s_ the projected surface area of the membrane, and *n* the number of cycles. We estimated the values of *V*_r_ and *A*_s_ as approximately 2.5 × 10^−9^ m^3^ and 0.0042 m^2^, respectively (Experimental Section). The water‐rich permeate flux was assumed constant (*J*_w_ = 289 L m^−2^ h^−1^, see Figure 5). We determined the value of κ as 2.77 s^−1^ by fitting ln ((1 −*η*/100)^−1^) = *κτ*. Figure 6c demonstrates plots of the measured and the fitted photocatalytic degradation of TCDD as a function of permeation cycles. It shows that the photocatalytic degradation (η) of TCDD reaches >99% after *n* = 298 cycles. Further, the predictions using the first order kinetic model match well with the experimental data. This verifies that a decrease of TCDD concentration in the water‐rich permeate upon repeated filtration cycle is due to photocatalysis rather than adsorption on the membrane surface. We also demonstrated that our separation methodology photocatalytically degrades permethrin (a pesticide toxin) in the water‐rich permeate (Section S9, Supporting Information).

## 3 Conclusion

We have developed an in‐air superhydrophilic and underwater superoleophobic membrane by utilizing a thermally sensitized stainless steel mesh spray coated with photocatalytic Fe−TiO_2_ nanoparticles. It was demonstrated that post‐annealing increases the adhesion force of Fe−TiO_2_ nanoparticle to the membrane by the formation of a fusion‐induced bridge between them. We have engineered an apparatus that enables continuous separation of surfactant‐stabilized oil‐in‐water emulsion and in situ photocatalytic degradation of the dissolved organic contaminants in the water‐rich permeate upon irradiation of visible light on the membrane surface. We showed that our membrane recovers the flux of the water‐rich permeate nearly 99% upon irradiation of visible light while it was continuously separating surfactant‐stabilized oil‐in‐water emulsion. Finally, we demonstrated complete separation of surfactant‐stabilized oil‐in‐water emulsion and photocatalytic degradation of toxins such as dioxin and permethrin by utilizing our apparatus. We envision that our separation methodology can offer a wide range of potential applications including petroleum refining, wastewater treatment, and clean‐up of oil spills.

## 4 Experimental Section

##### Synthesis of Fe−TiO_2_ Nanoparticles

A 50 mL of titanium butoxide (Ti(BuO)_4_) was added dropwise to a 65 mL solution of sodium hydroxide (NaOH, 1 m in DI water) and stirred for 2 min. The pH of the solution was measured as 8.3 by utilizing the pH meter (Mettler Toledo S220). Subsequently, ferric nitrate (Fe(NO_3_)_3_) solution in DI water (0.3 m) was added dropwise to the solution of Ti(BuO)_4_ and NaOH. Please note that the molar ratio of Ti and Fe were 100:0.25, 100:0.5, 100:1, and 100:5. The solution was then heated at 200 °C with vigorous stirring for 600 min in nitrogen‐purged environment. The precipitates were collected by centrifugation^[^ ^61^ ^]^ and subsequently thoroughly rinsed several times with ethanol and DI water. The obtained Fe−TiO_2_ nanoparticles were dried in a vacuum oven for 24 h at room temperature (22 °C). Finally, Fe−TiO_2_ nanoparticles were calcinated at 400 °C with a heating rate of 10 °C min^−1^ for 180 min.

##### Thermal Sensitization of Stainless Steel Mesh

Thermal sensitization of stainless steel mesh (9.0 × 4.7 cm, nominal pore size = 2 µm) was conducted utilizing a furnace in air (Thermo Scientific Thermolyne Benchtop Muffle furnace). The sensitizing temperature was 600, 800, 1050, and 1360 °C.

##### Spraying Fe−TiO_2_ Nanoparticles onto the Thermally Sensitized Stainless Steel Mesh

A 0.05 mg mL^−1^ suspension of Fe−TiO_2_ in ethanol was sprayed (iWata spray gun) onto the thermally sensitized stainless steel mesh. The spraying air pressure and a distance were maintained at 29 psi and 20 cm, respectively. Please note that all meshes were sprayed with the same amount of Fe−TiO_2_ (≈5 mg).

##### Post‐Annealing

Fe−TiO_2_ spray‐coated stainless steel meshes were annealed in air using a furnace at the same temperature as the sensitizing temperature (i.e., 600, 800, 1050, and 1360 °C) for 60 min.

##### Measuring the Intensity of Incident Light

The intensity of the incident light on the membrane surface was measured by utilizing a photometer (Fisherbrand Traceable Dual‐Display Lightmeter). The photometer was placed underneath the cross‐flow cell top cover and irradiated by a visible light source from the same distance (≈10 cm) at which the membrane was irradiated during separation.

##### Determining the Nominal Pore size of Membrane

We utilized the filter retention analysis^[^ ^50^ ^]^ to determine the nominal pore size of membranes. Silica (SiO_2_) particles with various diameters were sequentially fed to the membrane in the order of the lowest to the highest. The proportion of the particles retained on the membrane was calculated for each size according to, %*R* = *M*_R_/*M*_T_, where *M*_R_ and *M*_T_ are the mass of SiO_2_ retained on the membrane and the total mass of that introduced to the membrane, respectively. The diameter of SiO_2_ was assigned as the nominal pore size of the membrane if %*R* exceeds 50% for that particular size. We used monodisperse SiO_2_ with diameters of 120, 150, 200, 300, 400, 500, 600, and 750 nm. SiO_2_ suspensions were prepared in ethanol with a concentration of 50 mg mL^−1^. The %*R* was measured as 54, 61, 59, and 72% with SiO_2_ possessing a diameter 200, 400, 500, and 750 nm for membranes subjected to sensitized at *T*_s_ = 1360, 1050, 800, and 600 °C, respectively. Therefore, 200, 400, 500, and 750 nm were assigned as the nominal pore size of membranes obtained at *T*_s_ = 1360, 1050, 800, and 600 °C, respectively.

##### Tape Peel Off Test^[^ ^51^ ^]^

The standard Tape Peel off test was conducted by utilizing the Elcometer 99 standard ASTM tape. The tape was applied to the membrane surface and then removed. The test was repeated 10 times. The mass of the membrane before and after the Tape Peel off tests was measured by utilizing a Mettler Toledo XS105 DU balance with precision down to 0.01 mg. The percentage mass loss of Fe−TiO_2_ nanoparticles was calculated by comparing the membrane's mass before and after the Tape Peel off test.

##### Scanning Electron Microscopy

The membrane surface texture and morphology were characterized by using field emission scanning electron microscopy (FE‐SEM, FEI Versa 3D DualBeam) at an accelerating voltage of 10 kV. To prevent charging, all surfaces were sputter coated with a thin layer of gold (≈2–3 nm).

##### Estimation of the Feature Size of the Mesh after Thermal Sensitization

We determined the average feature size of the thermally sensitized mesh by analyzing the size distribution of the ImageJ software on the SEM images.

##### X‐Ray Diffraction Measurement

The crystal structure of Fe‐doped TiO_2_ was studied by powder X‐ray diffraction (XRD) with a PANalytical Model X'Pert PRO diffractometer with Cu Kα radiation (*k* = 1.54 Å) by scanning at a rate of 2° (2θ) min^−1^.

##### X‐Ray Photoelectron Spectroscopy

X‐ray photoelectron spectroscopy (XPS) was performed by a Phi Versaprobe II using monochromatic source Mg Kα.

##### Optical Profilometry

The root mean square (RMS) surface roughness of membranes was measured using optical profiler (Veeco Wyko NT 1100) at a scan rate of 50 nm s^−1^. The scanned area was 5 × 5 µm.

##### Determining Fe−TiO_2_ Nanoparticle Size

The average size of Fe−TiO_2_ nanoparticle was measured using dynamic light scattering (DLS) (Zetasizer Nano ZS ZEN3600, Malvern, UK) with a red laser (633 nm). A suspension of Fe−TiO_2_ (1 mg mL^−1^) was prepared in water followed by vigorous stirring by ultrasonication.

##### Engineering a Continuous Separation Apparatus

The continuous separation apparatus consisted of a feed storage tank, a pump (2SF22SEEL WEG industries), a differential pressure gauge (OMEGA DPG409‐500DWU), visible light source (Sugarcube ultra LED, 13.1 W), a permeate tank, and the cross‐flow cell (Sterlitech, CF042A). The cross‐flow cell consisted of two transparent acrylic counterparts sandwiching the membrane in between. The effective surface area of the membrane was 0.0042 m^2^. The membrane surface was irradiated by visible light with an intensity of 150 mW cm^−2^. Please note that the cross‐flow cell was thoroughly rinsed with DI water and ethanol after each experiment.

##### Oil‐in‐Water Emulsion

An oil‐in‐water emulsion was prepared by vigorously mixing *n*‐hexadecane and DI water with a 5:95 v:v *n*‐hexadecane:water ratio. The emulsion was stabilized by SDS with 0.3 mg mL^−1^ of emulsion. The emulsion dissolved with TCDD (or permethrin) was prepared by mixing TCDD solution (0.01 mg mL^−1^ in methanol) or permethrin in ethanol (0.01 mg mL^−1^ in ethanol). The resulting emulsion contained 10 ppm of TCDD (or permethrin) in *n*‐hexadecane‐in‐water emulsion.

##### Measuring the Underwater Adhesion Force of an Oil Droplet to the Membrane

A high precision microelectromechanical system (Data‐Physics DCAT 11, Germany) was utilized to measure the adhesion force of an oil droplet to the membrane. A piece of membrane (2 × 2 cm) was fully submerged in a DI water bath. A droplet of *n*‐hexadecane (≈ 5 μL) was held by a metal needle tip submerged in water. The water bath was slowly moved upward at a constant speed of 0.1 mm s^−1^ until it contacted the oil droplet. The membrane was slowly moved downward until the oil droplet detached from the membrane surface at which point the adhesion force was recorded.

##### Thermogravimetric Analysis

TGA was performed using PekinElmer PYRIS 1 Thermogravimetric Analyzer. Approximately 0.01 mL of the water‐rich permeate was heated from room temperature to 110 °C at a rate of 5 °C min^−1^ followed by maintaining a constant temperature of 110 °C for 50 min. As the boiling points of water and *n*‐hexadecane are 100 and 287 °C, respectively, the weight loss of the water‐rich permeate was used to determine the separation efficiency.

##### Ultraviolet−visible Spectroscopy

UV−Vis spectrophotometry was performed by utilizing Thermo Evolution 600 UV/Visible Spectrophotometer at a scan speed of 240 nm min^−1^ with a data interval of 2 nm.

##### Estimation of Fe−TiO_2_ Nanoparticle Volume (*V*_r_)

We estimated the value of *V*_r_ by measuring the membranes mass before and after spray coating with Fe−TiO_2_ NPs. The value was then divided by the density of Fe−TiO_2_ (≈3940 mg cm^−3^).

## Conflict of Interest

The authors declare no conflict of interest.

## Author Contribution

G.K. designed the research. M.E., B.S., S.K., B.J., J.G., and K.H. performed experiments. M.E., B.S., D.L., and G.K. analyzed the data. M.E., D.L., and G.K. wrote the manuscript.

## Supporting information

Supporting Information
