# Supplementary material for: Selective Wettability Membrane for Continuous Oil−Water Separation and In Situ Visible Light‐Driven Photocatalytic Purification of Water
Source: Glob Chall. 2020 Apr 16;4(10):2000009. doi: 10.1002/gch2.202000009 (PMC7533845; doi:10.1002/gch2.202000009)
Supplement: Supplementary file 1 — Supporting Information [file GCH2-4-2000009-s001.pdf]

Copyright WILEY-VCH Verlag GmbH & Co. KGaA, 69469 Weinheim, Germany, 2020.

Supporting Information

for *Global Challenges,* DOI: 10.1002/gch2.202000009

Selective Wettability Membrane for Continuous Oil#Water Separation and In Situ Visible Light-Driven Photocatalytic Purification of Water

Mohammadamin Ezazi, Bishwash Shrestha, Sun-I. Kim, Bora Jeong, Jerad Gorney, Katie Hutchison, Duck Hyun Lee, and Gibum Kwon**

Copyright WILEY-VCH Verlag GmbH & Co. KGaA, 69469 Weinheim, Germany, 2018.

Supporting Information

Selective Wettability Membrane for Continuous Oil-Water Separation and In Situ Visible Light-Driven Photocatalytic Purification of Water

Mohammadamin Ezazi, Bishwash Shrestha, Sun-I Kim, Bora Jeong, Jerad Gorney, Katie Hutchison, Duck Hyun Lee, and Gibum Kwon**

Section 1. Analysis of crystal structure, elemental composition, and size of Fe-TiO_2 nanoparticles

X-ray diffraction (XRD) patterns of neat titania (TiO_2) and iron (Fe)-doped titania (TiO_2) nanoparticles (Fe-TiO_2) with various ferric ion (Fe^{3+}) dopant mol% are shown in **Figure S1**a. The characteristic peaks at 2θ = 25.26°, 37.96°, 48.15°, 54.06°, 55.13°, 62.75°, 69.04°, 70.66°, and 75.39° correspond to the anatase phase with lattice planes (101), (103), (200), (105), (211), (204), (116), (220), and (107), respectively. Further, the characteristic peak at 2θ = 27.61° corresponds to the rutile phase with a lattice plane (121).[1] The intensity of these characteristic peaks for anatase and rutile phases decreases as the mol% of Fe^{3+} increases. This has been attributed to the perturbation that occurs in anatase and rutile structures after doping with Fe^{3+}.[2,3] **Figure S1**b shows the x-ray photoelectron spectroscopy (XPS) spectrum of Fe-TiO_2 with 1 mol% Fe^{3+}. It clearly shows Ti, O, and Fe peaks. The peaks at binding energies of 458 eV and 464 eV correspond to Ti $2p_{3/2}$ and Ti $2p_{1/2}$ spin–orbital splitting photoelectrons, respectively. These values are in good agreement with values of titanium ion (Ti^{4+}).[4,5] The O 1s peak at 528 eV corresponds to the lattice oxygen of TiO_2.[6] The Fe 2p spectrum exhibits peaks at 709 and 722 eV which corresponds to Fe $2p_{3/2}$ and Fe $2p_{1/2}$, respectively.[4] This suggests that the Fe substituted Ti^{4+} lattice possesses the Fe^{3+} chemical state. **Figure S1**c shows the number size distribution of Fe-TiO_2 nanoparticles synthesized in this work. Dynamic light scattering (DLS) data show that the average size is between 20±2 nm.

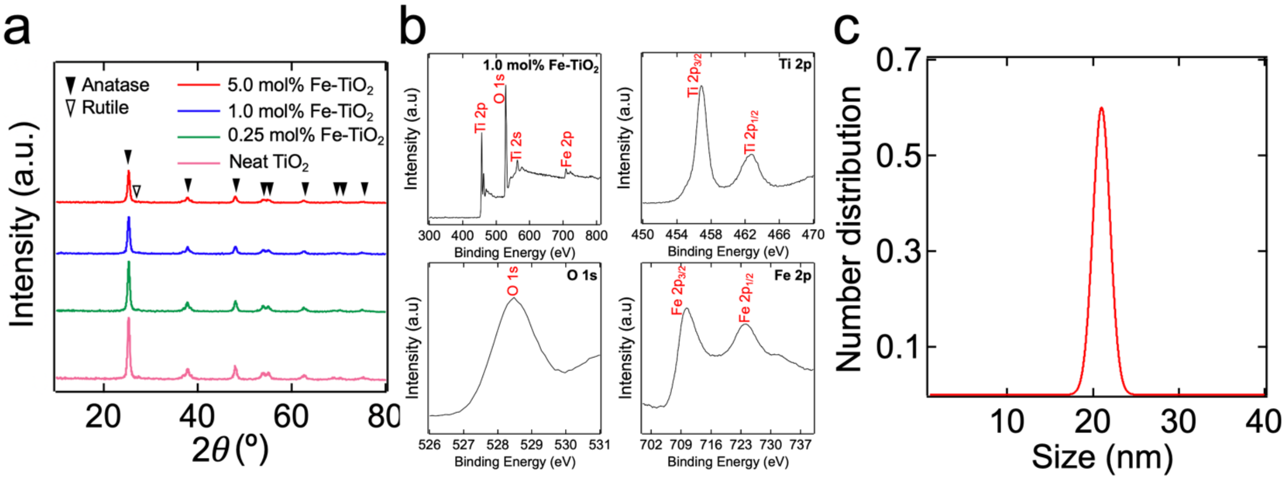

Figure S1. (a) XRD patterns of $Fe\text{-}TiO_2$ with varied Fe^{3+} dopant concentration. (b) XPS spectrum of $Fe\text{-}TiO_2$ with 1 mol% Fe^{3+} dopant with core level spectra of characteristic Ti 2p, O 1s, and Fe 2p. (c) The number size distribution of $Fe\text{-}TiO_2$ nanoparticles determined by DLS.

Section 2. Effects of visible light intensity on photocatalytic degradation kinetics of solvent blue dye in water

We evaluated the photocatalytic degradation activity of $Fe\text{-}TiO_2$ upon visible light irradiation with varied intensity. $Fe\text{-}TiO_2$ nanoparticles (1.0 mol%) were dispersed in solvent blue dye solution (0.02 mg mL^{-1} in deionized (DI) water) with a concentration of 0.1 mg mL^{-1}. 10 mL of the dispersion was poured in a transparent glass container equipped with magnetic stirrer. The visible light guide (Experimental Section in the main text) was submerged into the container to irradiate the dispersion. A small quantity (2 mL) of the dispersion was taken at every 20 mins. It was centrifuged and filtered by filter paper followed by UV-Vis spectrophotometry. **Figure S2** demonstrates the photocatalytic degradation (η) defined as $\eta = (1 - C_n/C_o) \times 100$, where C_o and C_n are the initial concentration of solvent blue dye and that at time n, respectively as a function of irradiation time. As the intensity increases, the photocatalytic degradation of solvent blue rapidly increases at a given irradiation time. At Intensity = 150 mW cm^{-2}, a complete photocatalytic degradation was observed at ≈ 180 mins. At a higher irradiation intensity such as Intensity = 200 and 250 mW cm^{-2}, a complete photodegradation was observed at much shorter time of approximately 160 and 140 mins, respectively.

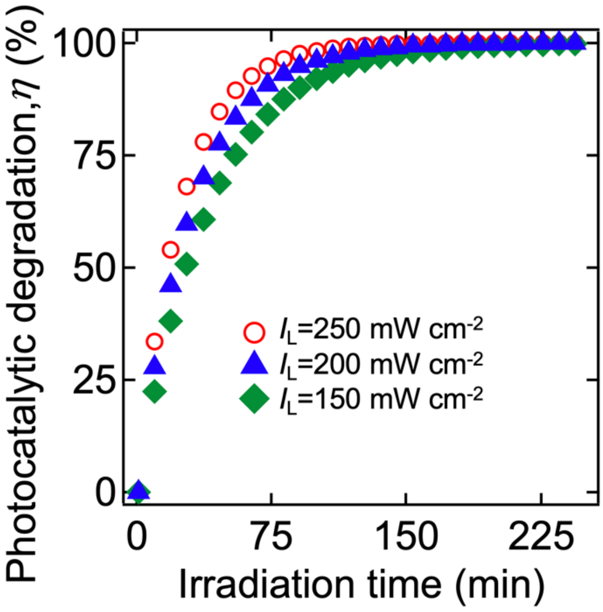

Figure S2. The photocatalytic degradation of solvent blue dye in water by 1.0 mol% Fe-TiO_2 nanoparticles at various irradiation light intensities as a function of irradiation time.

Section 3. Effect of sensitizing time (t_s) on the surface texture of membrane

We evaluated the effect of thermal sensitizing time on the evolution of the surface texture. **Figure S3** demonstrates the SEM images of a membrane sensitized at 800 °C for various sensitizing times (t_s). The non-uniformity of surface texture is apparent when t_s<900 mins (**Figure S3**a). When t_s≥900 mins, the surface is covered homogenously by a uniform size of features and homogeneously covered (**Figure S3**b and **S3**c).

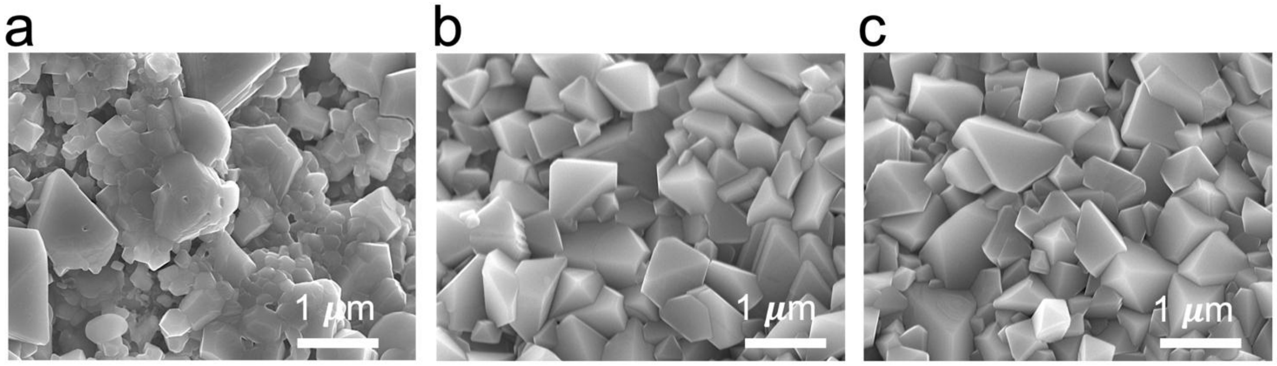

Figure S3. SEM images of a membrane surface sensitized at 800 °C for varied sensitizing times, (a) t_s<900 mins, (b) t_s=900 mins, and (c) t_s>900 mins.

Section 4. Estimating the solid-oil interface area fraction submerged in water

An oil contact angle on a rough surface (e.g., membrane) submerged in water (θ_2) can be described by the Cassie-Baxter relation given by[7,8]:

(S1) $\cos\theta_2 = f\cos\theta_1 + f - 1$

where θ_1 is the advancing contact angle for oil (e.g., n-hexadecane) on a smooth surface submerged in water. f is the area fraction of the solid-oil. Equation S1 can be rearranged and written in terms of f as follows:

(S2) $f = (1 + \cos\theta_2)/(1 + \cos\theta_1)$

We measured θ_1 = 123°. The estimated values of f of membranes sensitized at varied temperatures are listed in **Table S1**.

Table S1. Estimated values of the f for membranes sensitized at varied sensitizing temperatures

Membranes	θ_2 [°]	f
Sensitized at 600 °C	155	0.20
Sensitized at 800 °C	158	0.16
Sensitized at 1050 °C	160	0.13
Sensitized at 1360 °C	151	0.28

Section 5. Underwater roll-off angles and adhesion force for various oils on the membranes

Figure S4 shows the measured underwater contact angles and adhesion force for various oils; n-hexane (γ_{lv} = 18.4 mN m^{-1}), n-heptane (γ_{lv} = 20.1 mN m^{-1}), n-octane (γ_{lv} = 21.6 mN m^{-1}), n-decane (γ_{lv} = 23.8 mN m^{-1}), n-dodecane (γ_{lv} = 25.3 mN m^{-1}), n-hexadecane (γ_{lv} = 27.5 mN m^{-1}), methyl oleate (γ_{lv} = 31.3 mN m^{-1}), and rapeseed oil (γ_{lv} = 35.7 mN m^{-1}) on the membranes sensitized at 600, 800, 1050, and 1360 °C. It clearly shows that the roll-off angles and oil adhesion force is lower for oils with higher surface tensions (γ_{lv}).

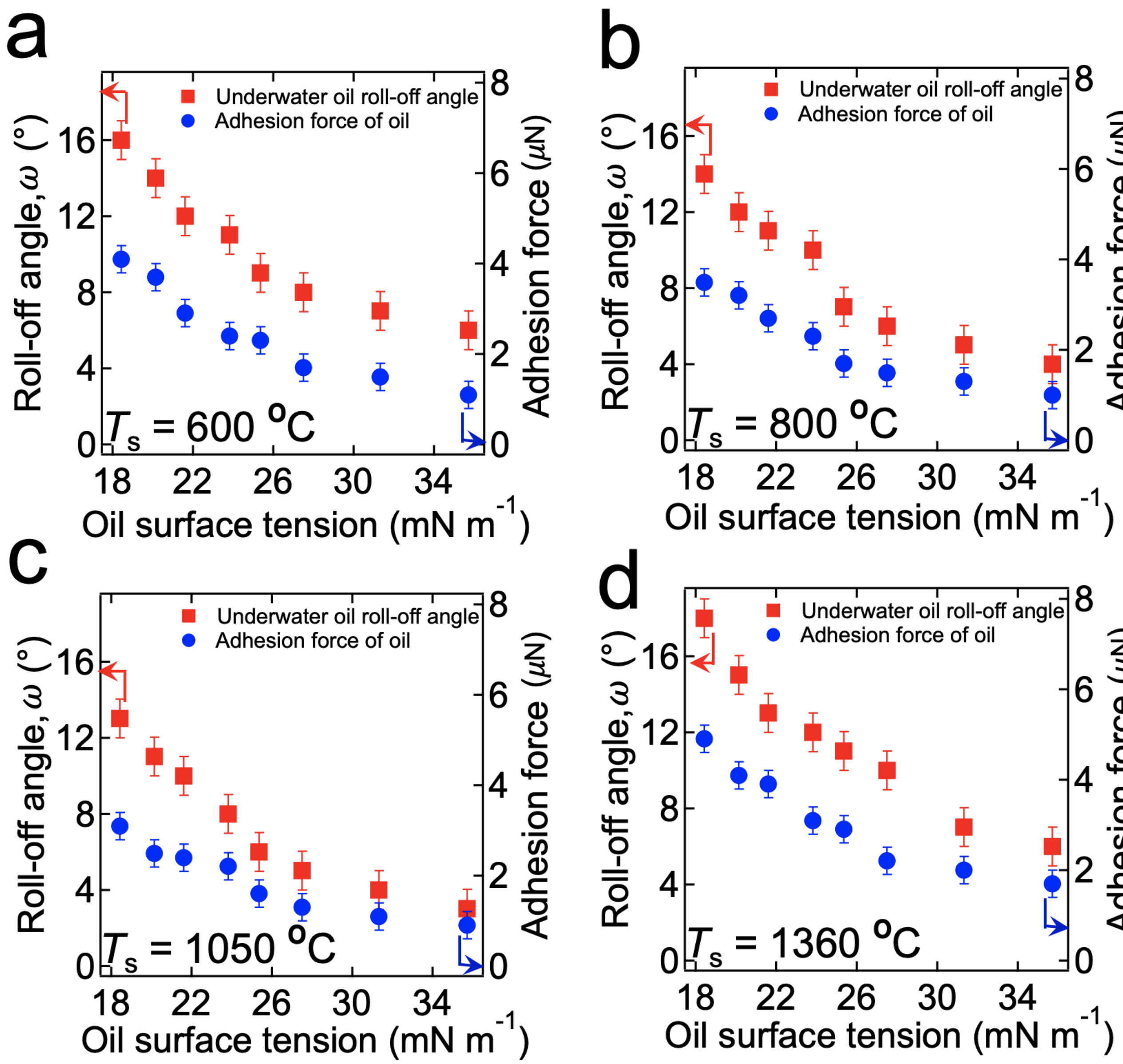

Figure S4. Measured underwater roll-off angles and the adhesion force for various oils on the membranes sensitized at varied temperatures, (a) T_s=600 °C, (b) T_s=800 °C, (c) T_s=1050 °C, and (d) T_s=1360 °C.

Section 6. Breakthrough pressure (P_b) of oil on the membranes

We measured the breakthrough pressure of oil on our membrane. After the membrane is prewetted by DI water for at least 60 mins, pure n-hexadecane was pumped out from the feed storage tank and introduced to the membrane with a transmembrane pressure (TMP) of 0.1 psi. Then, the TMP was gradually increased until n-hexadecane started to flow through the membrane and collected in the permeate tank. This pressure was considered the breakthrough pressure (P_b). The breakthrough

pressures for membranes sensitized at T_s = 600, 800, 1050, and 1360 °C were obtained as 2.2±0.2, 2.4±0.1, 2.5±0.3, and 2.1±0.3 psi, respectively.

Section 7. Size distribution of the dispersed oil droplet in SDS-stabilized oil-in-water emulsion

We determined the size distribution of the dispersed oil droplets in the SDS-stabilized oil-in-water emulsion by utilizing dynamic light scattering (DLS). **Figure S5** shows that the average size of oil droplets is 1.6±0.6 μm.

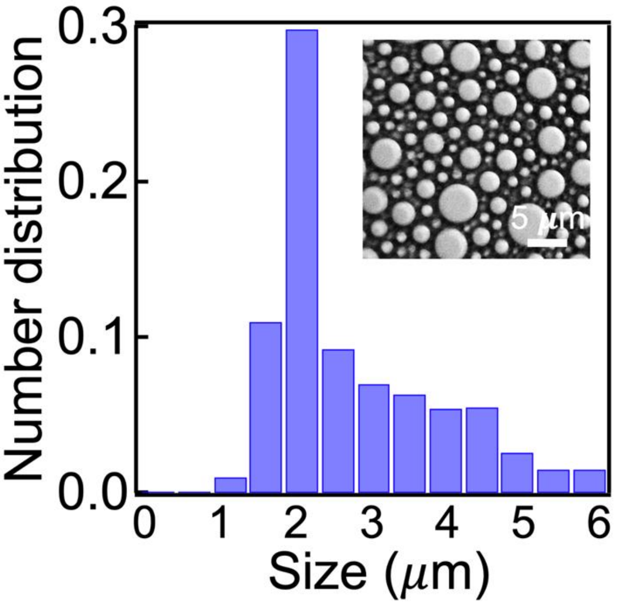

Figure S5. The number size distribution of the dispersed oil droplets in SDS-stabilized n-hexadecane-in-water emulsion. The inset shows a representative optical microscopy image of the oil droplets.

Section 8. UV-Vis absorption spectra and the concentration calibration curve for TCDD

Figure S6a and **S6**b show the UV-Vis absorption spectra of TCDD in water with varied concentrations and the calculated area under the curve, respectively. We calculated the area underneath the UV-Vis absorption spectra of TCDD aqueous solutions and the water-rich permeates via numerical integration by utilizing a code developed in MATLAB:

```
clc;
close all;
clear all;
data = xlsread('C:\Users\UV-Vis absorption spectrum.xlsx');
x=data(:,1);
y=data(:,2);
Area underneath UV-Vis = trapz(x,y)”
```

We calculated the area as 0.21, 1.02, 4.08, 6.93, 11.62, and 18.64 for TCDD dissolved water with concentrations of 0.1, 0.5, 2.0, 3.5, 6.0, and 10.0 ppm, respectively.

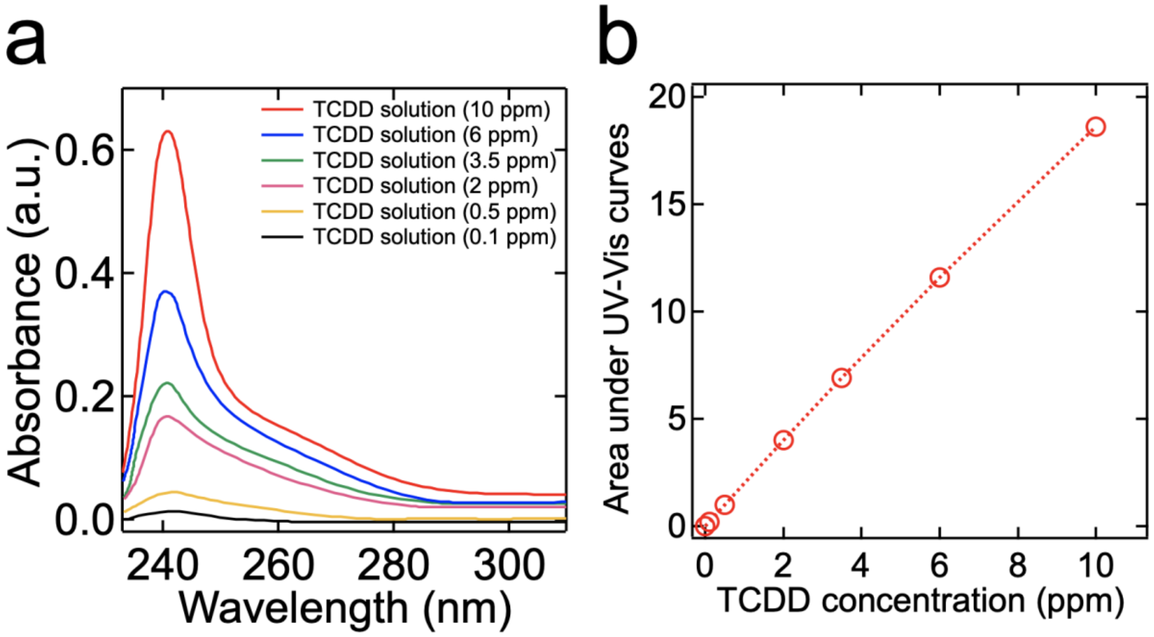

Figure S6. (a) UV-Vis absorption spectra of TCDD in water with varied concentrations, and (b) the calculated area under the UV-Vis absorption spectra.

Section 9. Photocatalytic degradation of Permethrin

Permethrin (3-phenoxybenzyl 3-(2,2-dichlorovinyl)-2,2-dimethylcyclopropanecarboxylate) is a pyrethroid-based pesticide.[9] We chose permethrin as a model toxin because it is categorized by Environmental Protection Agency (EPA) as a likely human carcinogen when ingested.[10] Photocatalytic degradation of permethrin by highly oxidative hydroxyl radicals ($OH^{\bullet}$) can cause nearly complete degradation by aromatic ring cleavage causing the formation of carbon dioxide (CO_2).[11] Similar to TCDD experiments in the main text, we conducted continuous oil-water separation and *in situ* photocatalytic degradation of permethrin.

Figure S7 demonstrates the change of UV-Vis absorption spectra of permethrin as a function of permeation cycles. The intensity of the characteristic absorption peak ($\lambda \approx 209$ nm)[11] of permethrin decreases with a number of permeation cycles and eventually disappears when n = 162. The quantitative analysis was conducted by utilizing the calibration curves created by calculating the area underneath the absorption spectra (**Figure S8**a and **S8**b). We calculated the area underneath the UV-Vis absorption spectra of permethrin dissolved water as 0.85, 4.25, 17.02, 21.08, 34.51, and 87.06 with concentrations of 0.1, 0.5, 2.0, 2.5, 4.0, and 10.0 ppm, respectively. We utilized the same MATLAB code shown in SI Section 8. **Figure S9** shows the photocatalytic degradation (η) as a function of permeation cycles which is defined as $\eta = (1 - C_n/C_o) \times 100$. Here C_o and C_n are the concentrations of permethrin in the feed and that in the water-rich permeate, respectively, after the nth cycle. We described the photocatalytic degradation of permethrin dissolved in the water-rich permeate during permeation by the first order kinetics (equation 1 and 2 in the main text). We determined the value of κ as 5.57 s^{-1} by calculating the slope of the fitting line to the equation $\ln((1 - \eta/100)^{-1}) = \kappa\tau$. **Figure S9** shows that the photocatalytic degradation (η) of permethrin reaches > 99% after n = 162 cycles. Further, the predictions using the kinetic model match well with the experimental data verifying that the decrease of the permethrin concentration in the water-rich permeate is primarily due to photocatalytic degradation instead of adsorption.

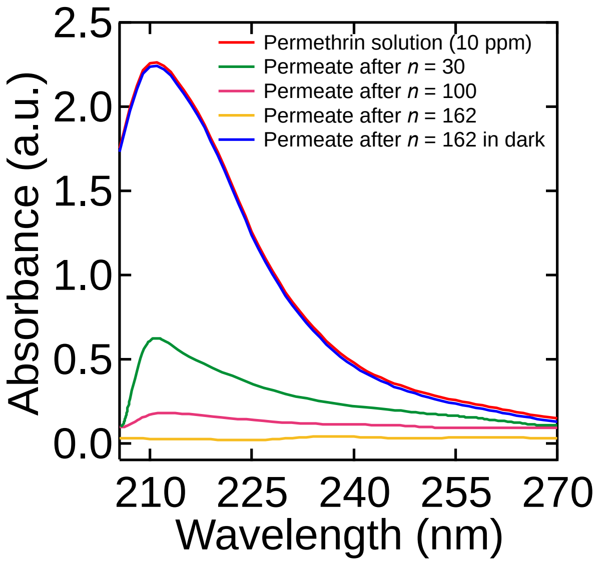

Figure S7. UV-Vis absorption spectra of permethrin dissolved in the water-rich permeate subjected to visible light-driven photocatalytic degradation as a function of repeated permeation cycles.

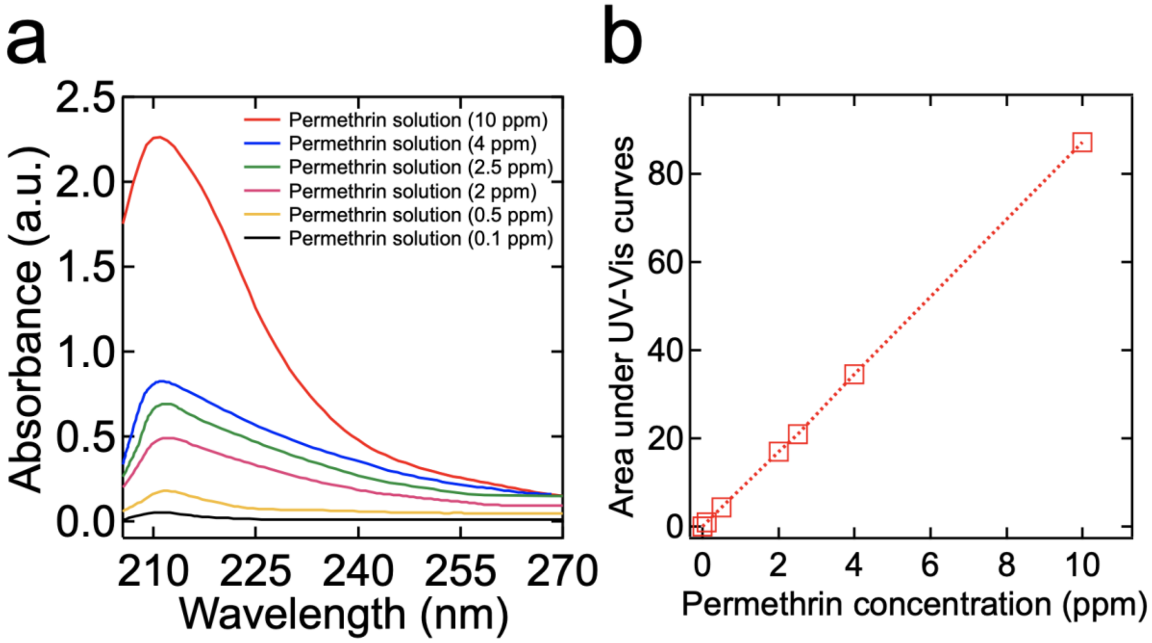

Figure S8. (a) UV-Vis absorption spectra of permethrin in water with varied concentrations and (b) the calculated area under the UV-Vis absorption spectra.

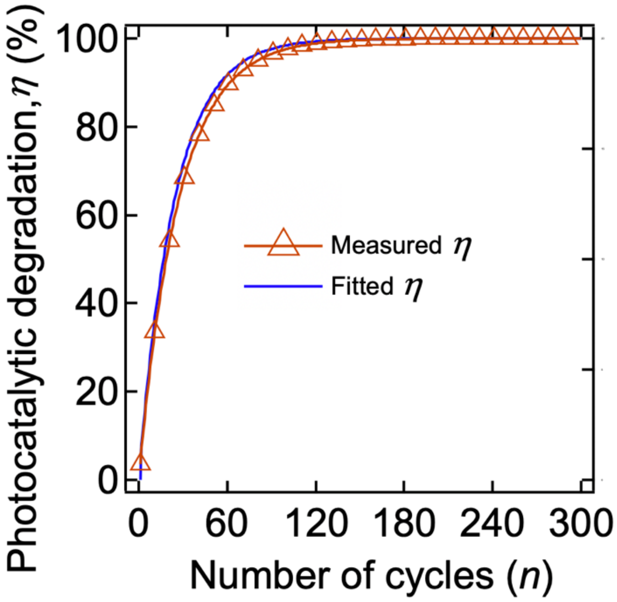

Figure S9. Plots of the measured and fitted photocatalytic degradation of permethrin as a function of permeation cycles.

References

[1] Y. Ding, Y. Wang, L. Zhang, H. Zhang, C. M. Li, Y. Lei, *Nanoscale* **2011**, *3*, 1149.
[2] X. Yang, C. Cao, L. Erickson, K. Hohn, R. Maghirang, K. Klabunde, *Appl. Catal., B* **2009**, *91*, 657.
[3] X. Wang, J.-G. Li, H. Kamiyama, M. Katada, N. Ohashi, Y. Moriyoshi, T. Ishigaki, *J. Am. Chem. Soc.* **2005**, *127*, 10982.
[4] T. Sun, E. Liu, J. Fan, X. Hu, F. Wu, W. Hou, Y. Yang, L. Kang, *Chem. Eng. J.* **2013**, *228*, 896.
[5] C. Wagner, W. Riggs, L. Davis, J. Moulder, G. Muilenberg, *Eden Prairie, MN* **1979**, 38.
[6] S. Sood, A. Umar, S. K. Mehta, S. K. Kansal, *J. Colloid Interface Sci.* **2015**, *450*, 213.
[7] A. Cassie, S. Baxter, *Trans. Faraday Soc.* **1944**, *40*, 546.
[8] M. Liu, S. Wang, Z. Wei, Y. Song, L. Jiang, *Adv. Mater.* **2009**, *21*, 665.
[9] S. C. Schimmel, R. L. Garnas, J. M. Patrick Jr, J. C. Moore, *J. Agric. Food Chem.* **1983**, *31*, 104.
[10] U. S. E. P. Agency, *EPA Washington, DC*, **2006**.
[11] H. Hidaka, K. Nohara, J. Zhao, N. Serpone, E. Pelizzetti, *J. Photochem. Photobiol., A* **1992**, *64*, 247.
